# The impact of the SARS-CoV-2 pandemic on financial markets: a seismologic approach

**DOI:** 10.1007/s10479-021-04115-y

**Published:** 2021-05-14

**Authors:** Alessandro Spelta, Nicolò Pecora, Andrea Flori, Paolo Giudici

**Affiliations:** 1 Department of Economics and Management, University of Pavia, Via San Felice, 5, 27100 Pavia, Italy; 2 Department of Economics and Social Sciences, Catholic University, Via Emilia Parmense, 84, 29122 Piacenza, Italy; 3 Department of Management, Economics and Industrial Engineering, Polytechnic of Milan, Via Lambruschini, 4/B, 20156 Milan, Italy

**Keywords:** SARS covid-2, Financial markets, Omori law, Agent-based modeling, G15, G18, G41

## Abstract

This work investigates financial volatility cascades generated by SARS-CoV-2 related news using concepts developed in the field of seismology. We analyze the impact of socio-economic and political announcements, as well as of financial stimulus disclosures, on the reference stock markets of the United States, United Kingdom, Spain, France, Germany and Italy. We quantify market efficiency in processing SARS-CoV-2 related news by means of the observed Omori power-law exponents and we relate these empirical regularities to investors’ behavior through the lens of a stylized Agent-Based financial market model. The analysis reveals that financial markets may underreact to the announcements by taking a finite time to re-adjust prices, thus moving against the efficient market hypothesis. We observe that this empirical regularity can be related to the speculative behavior of market participants, whose willingness to switch toward better performing investment strategies, as well as their degree of reactivity to price trend or mispricing, can induce long-lasting volatility cascades.

## Introduction

As the spread of the novel coronavirus (SARS-CoV-2) is causing a significant economic and financial burden in most countries of the world, financial economists, credit rating companies and country risk experts have scrambled to rearrange their assessments in the light of the unprecedented geo-economic challenges posed by the healthcare crisis.

On Monday 24th February 2020, the Dow Jones Industrial Average and FTSE 100 indices dropped more than 3%, as the SARS-CoV-2 outbreak spread worsened substantially outside China over the previous weekend. This came after the benchmark market indices sharply decreased in continental Europe following steep declines across Asia. The DAX, CAC40 and IBEX35 fell by about 4% and the FTSE-MIB tumbled by over 5%. On February 27th, the US stock market indices, including the NASDAQ-100, the S&P 500 Index and the Dow Jones, posted their sharpest falls since 2008, with the Dow Jones falling 1.191 points, its largest one-day drop since the 2008 financial crisis. Following a second week of turbulence, on March 6th, most of the stock markets worldwide closed down, while the yields on 10-year and 30-year U.S. Treasury securities fell to new record lows under 0.7% and 1.26% respectively. To face the epidemic impact, central banks and governments on the two sides of the Atlantic have reacted with massive recovery plans. The US Federal Reserve lowered its benchmark rate purchasing bonds on both the primary and secondary markets, also reactivating its currency-swap lines with other central banks, while the European Central Bank abandoned its rules for limiting sovereign and corporate bond purchases, launching a Pandemic Emergency Purchase Program, among other measures.

Against this background, such a financial disruption motivates our study on the volatility dynamics induced by SARS-CoV-2 related news on the major financial markets in Europe and US, during the period ranging from January to April 2020, as we deem this period refers to an unprecedented and unique stage in the history of financial markets. SARS-CoV-2 is, in fact, an exclusive event in terms of its global reach as a pandemic. While the exact global economic and financial consequences are not yet completely definite, financial markets have already responded with dramatic movements. The 2020 coronavirus stock turmoil which began on February 2020 and ended on April constituted a major and sudden crisis hitting stock markets globally. To show how timely intervention from political authorities impacted on stock indices through stimulus announcements, we focus on some relevant market-capitalized indices as illustrative examples and, among them, we select indices referring to economies heavily affected by the first wave of the pandemics outbreak. For instance, UK, Spain, France, Germany and Italy account for more than 65% of the total cases registered in Europe according to the WHO report^1^ of April 20th, while in the same period the United States, with 723,605 confirmed cases, were already the most hardly hit country worldwide.^2^ For these reasons, we present and discuss the application of our proposed framework on a parsimonious list comprising the aforementioned countries, which is instrumental to analyze heterogeneous responses of SARS-CoV-2 related news across different financial markets of relevant economies.

Specifically, our work analyzes the impact of socio-economic and political news, as well as of financial stimulus disclosures, on the volatility of the reference stock markets of the United States (S&P500), the United Kingdom (FTSE100), Spain (IBEX35), France (CAC40), Germany (DAX) and Italy (FTSE-MIB). We select major events related to the evolution of the epidemic between January 2020, when the first cases manifested, and the end of April 2020, thus including all the lockdown and mobility restriction measures, the announcements of economic aid packages (both from single countries and of supranational authorities), and the lockdown lifting decisions (see Appendix, Tables 1, 2, for details on SARS-CoV-2 related news). These events highlight the importance of considering the different types of policies that have been worldwide announced to cope with the unprecedented shocks that hit most of the economies and financial markets around the world (see Cheng et al. 2020).

To investigate the effects of pandemic related news on financial volatility, we employ concepts developed in the field of seismology (see Omori 1894; Utsu 1961), deriving parallels between energy dissipation and information cascades. Omori law was originally proposed by F. Omori over a century ago and, since then, it has been recognized as one of the empirical laws in seismology. Generally speaking, it states that the frequency of after-shocks decays decreases in time approximately by the reciprocal of time following the main shock. Utsu (1961) showed that the decay rate of after-shocks was somewhat faster than that suggested by the original Omori formula.

In the present work, we quantify market efficiency in processing SARS-CoV-2 related news by means of the estimated Omori power-law exponents and we relate these empirical regularities to investors’ behavior through the lens of a stylized Agent-Based financial market model. This approach turns out to be very suitable for examining the nonlinear dynamics that takes place among the interacting players populating a financial market, and that cannot be grasped by only considering aggregate variables, such as asset prices or returns. Thus, one has to look into the detailed interactions among the market’s participants of an unstable financial systems. In so doing, we can link what is observed at macro-level with a precise description of the micro-level relationships, at the origin of the propagation of financial instability. We remark that a similar viewpoint has been adopted by Gao and Hu (2014) who examined the income structures of different sectors in a selected economy with an “anatomical approach” and showed that the losses can be modeled by an Omori law distribution, suggesting that instability propagates from the crisis initiating sector to other sectors. The common conclusion that can be drawn from our similar macro-to-micro approach is that aggregated variables are not always capable to grasp the dynamics generated by market players in unstable economies, and thus the inclusion of micro and behavioral features bears relevance for the explanation of the volatility behavior that follows major shocks related to an extreme event, such as the SARS-CoV-2 pandemic.

More in detail, we aim at recognizing whether external news related to the epidemic evolution could induce significant “after-shocks” (as well as “pre-shocks”) effects in the system, by producing dynamic relaxation in the volatility behavior, in line with the cascade effect of energy propagation which follows an earthquake (see, e.g., Lillo and Mantegna 2003; Weber et al. 2007; Petersen et al. 2010a, b). Then, we provide a behavioral interpretation of the financial consequences generated by the SARS-CoV-2 pandemic by relating the observed market dynamics with agents’ investment attitudes.

We introduce a stylized Agent-Based model where market participants might generate volatility patterns, comparable to those of the observed market indices, by adjusting their investment strategies in face of external shocks. The dynamics of the model is driven by the trading behavior of heterogeneous speculators whose choice of a trading rule is endogenous and depends on the performance of the selected strategy and on the market momentum. The model considers a scenario in which a market-maker adjusts the asset price to the market excess demand generated by agents’ orders that are, in turn, set according to a technical or a fundamental trading rule. Agents update their strategy selection over time according to an evolutionary mechanism. The proportions of agents employing a given trading rule evolve according to a fitness measure, which is function of the past performance of each strategy (see e.g. Hommes 2002). The higher the fitness of a strategy is, the more market participants will adopt it. Thanks to the presence of the evolutionary selection between trading rules, we observe that periods of either turbulent or calm market dynamics may emerge and alternate. Within this framework, agents’ reactivity in adjusting orders to the price trend or to actual mispricing (e.g., due to news or announcements) plays a crucial role in reproducing the observed volatility patterns and the related Omori exponents, offering a theoretically support for the empirical dynamics based on the micro-behavior of market participants. The adoption of a stylized Agent-Based framework turns out to be relevant for the understanding of investors behavior at a micro-level reacting to different policy scenarios implemented at a macro-level, and to address the emerging challenges that are rapidly engulfing businesses affected by the pandemic. Thus, on the one hand, the choice of the seismologic approach is motivated by the fact that in the period following a large market crash, markets show long-lasting activities, which follow the Omori law. On the other hand, the long-lasting dynamics observed in the course of stock prices is often due to the interacting behavior of markets’ participants that can herd towards a common strategy and that lead the price to deviate from its fundamental value. This feature may cause crashes, and thus volatility outbursts, to be locally self-enforcing or dissipating.

The remainder of the paper is organized as follows. Section 2 contains a literature review related to the study of market volatility cascades and empirical regularities occurring after a financial crash. Section 3 presents the evidence of Omori relationship in the dataset we analyze. Section 4 provides an explanation of the empirical facts through the formalization and simulation of an Agent-Based model. Section 5 concludes.

## Literature review

Perturbations in asset prices induced by exogenous shocks are widely studied by economists, mathematicians, and physicists (see, e.g., Fama 1965; Ding et al. 1993; Mandelbrot 1997; Mantegna and Stanley 1999), with particular emphasis on the market dynamics generated by extreme events (see e.g. Danielsson et al. 2012; Adelfio et al. 2020). It is well recognized that several aspects play a key role in financial systems, such as the behavior of heterogeneous interacting agents and the non-equilibrium behavior in the processes that generate financial dynamics, as shown for instance in Spelta et al. (2020a, 2021) and Avdjiev et al. (2019). In this context, the Omori law, which describes the non-stationary phase observed after an earthquake, turns out to be instrumental for describing the dynamics of a financial system when it is pushed far away from its equilibrium state by the occurrence of an extreme event. In seismology, the occurrence of an earthquake main shock increases the likelihood of other subsequent after-shocks. Similarly, in the financial context, the occurrence of an exogenous shock on an asset price or return may increase the likelihood of other shocks. In particular, several papers (see, e.g., Lillo and Mantegna 2003, 2004; Petersen et al. 2010a, b; Selçuk 2004; Selçuk and Gençay 2006) have shown that a power-law tail describes quite well the dynamics of market volatility after a major financial shock. Based on statistical regularities of volatility cascades triggered by exogenous events, economists have employed the Omori law to study financial markets’ after-shocks dissipation.

Sornette et al. (1996) investigated the volatility dynamics of the S&P 500 index before and after the Black Monday of October 19th, 1987. They found that the implied market volatility after the crash has a power-law log-periodic decay rate behavior. Similarly, Lillo and Mantegna (2003, 2004) analyzed the decaying rate of volatility of the NYSE around the Black Monday crash and found that the number of volatility spikes above a certain threshold follows a power-law which is equivalent to the modified Omori law, as also reported in Siokis (2012b) who found that the distribution of market volatility before and after stock market crashes has a power-law relaxation consistent with the Omori law.

Selçuk (2004), employing stock market data from Argentina, Brazil, Hong Kong, Indonesia, Korea, Mexico, Philippines, Singapore, Taiwan and Turkey, is the first attempt which characterizes financial volatility after crashes in emerging stock markets through the Omori law. Other works, such as Selçuk and Gençay (2006), Weber et al. (2007) and Mu and Zhou (2008) applied the same technique to intraday data of several stock market indices, e.g. the Dow Jones and the Shanghai Stock Exchange Composite. In particular, Selçuk and Gençay (2006) analyzed the dynamics of Dow Jones Industrial Average (DJIA) during the period from September 19th, 1994 to October 16th, 2002. In this sample period two shocks occurred, one on October 8th, 1998 when the DJIA went down by 2.9% within 5 min without the trades being suspended, and another on January 3rd, 2001 when the DJIA went up by 2.6% in 5 min without the trades being suspended, the latter being the largest positive shock within the sample period. They concluded that the return and volatility distribution changed non-linearly and that in fact can be characterized by the Omori law. Weber et al. (2007) studied return time series during the after-shock periods in three different data sets. The first one contains the Black Monday crash, and also a smaller crash that happened on September 11th, 1986. The second covers Trade & Quotes of the year 1997 and focuses on the crash that happened on October 27th, 1997, while the third studies the 1 min return series of General Electric stock in the three months after 11 September 2001. Overall, they noticed that smaller sub-crashes were present and each of them also follow the Omori’s process, but on a smaller scale. Mu and Zhou (2008) examined the volatility dynamics of the Shanghai Stock Exchange Composite (SSEC) after large volatility shocks and found that for the SSEC the decay rate parameter was often higher than 1, implying a faster pace of convergence towards the normal state. Finally, Petersen et al. (2010a) investigated whether the FED announcements about interest rate changes may generate volatility outbursts that decay as a power-law, analogously to the Omori law, Petersen et al. (2010b), by studying the dynamics of the 531 most traded US stocks before and after 219 shocks, found a similar power-law behavior but for the rate of pre-shocks, while Siokis (2012a) applied the Omori law to study foreshocks and aftershocks related to the dynamics of the exchange rate market during the South-Asia crisis of 1997. Differently from these works, our analysis covers a much larger number of events and of different nature (being these related to political, financial or medical announcements) that hit different financial markets in the world. This allows us to compare how competing markets reacted when absorbing different types of shocks, and, eventually, it also helps us to understand how these markets were able to anticipate the occurrence of external and sudden news related to the pandemic evolution.

## The Omori law

To study the market volatility dynamics around the date $T_{s}$ of external shocks induced by SARS-CoV-2 related news, we analyze the rate of decay of large volatility fluctuations during 10 trading days following (and preceding) the day of each announcement.

### Methodology

We compute the market volatility $V_{i}(t)$ following previous researches that deal with similar topics (see, e.g., Weber et al. 2007; Petersen et al. 2010a, b; Nowak et al. 2011) as:^3^

1 $$\begin{aligned} V_{i}(t) = |\log (P_{i}(t)) - \log (P_{i}(t-1))| \end{aligned}$$

The binary volatility time series $n_{i}(t)$ for each market *i*, is calculated as:

$$\begin{aligned} n_{i}(t)= {\left\{ \begin{array}{ll} 1, V_{i}(t)\ge q_{i}\\ 0, V_{i}(t)< q_{i} \end{array}\right. } \end{aligned}$$

where the threshold *q* is defined as the value of the 85-th percentile of the volatility distribution of market *i*. This approach allows us to study the number of times volatility series exceeds a given threshold value. This investigation is analogous to the investigation of the number of after-shock earthquakes measured at time *t* after the main earthquake. Nonetheless, in order to provide robustness to our approach, in the Appendix we perform a sensitivity analysis employing different thresholds. Moreover, as shown by Lillo and Mantegna (2003), the power-law behavior of volatility decay is observed only for large thresholds. Hence, the Omori Law is valid only for large values of the *q* employed to determine the exponent.

We describe the response of different financial markets to the SARS-CoV-2 related announcements deriving a parallelism between the energy relaxation of after-shocks following the main earthquake, described by the Omori law (Omori 1894), and markets’ volatility cascade dynamics induced by external news. The Omori law states that the number of after-shock earthquakes per unit time, measured at time *t*, decays as a power-law. Analogously, the rate *N*(*t*) of large volatility events following a single perturbation at time $T_{s}$ is defined as:

2 $$\begin{aligned} N_{i}(|t-T_{s}|)\sim |t-T_{s}|^{-\beta _{N_{i}}} \end{aligned}$$

where the parameter $\beta _{N_{i}}$ represents the Omori power-law exponent, and

$$\begin{aligned}N_{i}(t)=\frac{1}{J}\sum _{j=1}^{J} n_{i,j}(t) \end{aligned}$$

is the average rate of high volatility occurrences produced by all the *J* events in the *i*-th financial market, with *j* referring to the single SARS-CoV-2 related announcement. We remark that averaging across different event dates allows for better statistical regression. To estimate the power-law relationship between large volatility fluctuations and displacement time, we focus on $\Phi _{i}(|t-T_{s}|)$, which represents the cumulative number of events above the threshold *q* at time *t*, namely:

3 $$\begin{aligned} \Phi _{i}(|t-T_{s}|)=\int _{T_{s}}^{t} N_{i}(|t'-T_{s}|)dt'\propto |t-T_{s}|^{1-\beta _{N_{i}}} \end{aligned}$$

In order to compare the market dynamics before (*Pre*) and after (*Aft*) the occurrence of external events, we investigate separately volatility patterns around $T_{s}$. This means to discriminate between $N^{Pre}_{i}(t|t<T_{s})$ and $N_{i}^{Aft}(t|t>T_{s})$. We define the displaced time as $\tau =|t-T_{s}|$, then we employ a linear OLS fit on a log-log scale to estimate the Omori power-law exponents $\beta ^{Pre}_{N_{i}}$ and $\beta ^{Aft}_{N_{i}}$.

**Fig. 1:**
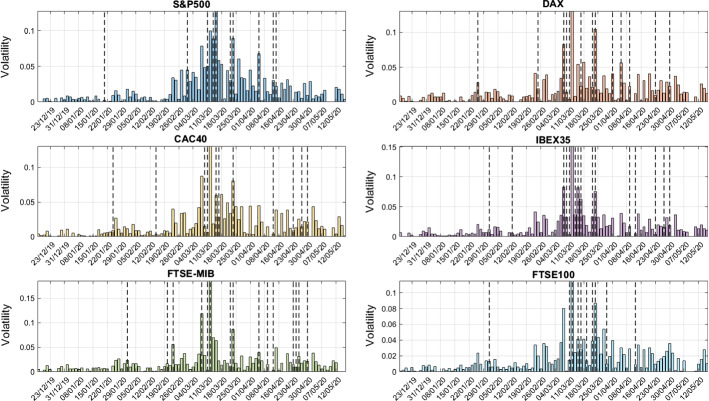
Volatility patterns for the selected market indices along with event dates. Column bars represent, for each country, the daily volatility of the reference index, computed as the absolute value of the returns derived from closure prices. The dashed black lines identify the dates of the relevant events, which mainly impacted the course of the national stock markets

### Empirical evidence of Omori relationship

We first analyze the volatility patterns of each reference stock index around the dates of SARS-CoV-2 related news. Figure 1 shows the volatility dynamics of the selected financial indices along with the dates of the selected SARS-CoV-2 related events, highlighted by the black dashed lines. The volatility patterns show correlated behaviors (see Appendix, Fig. 5) with a lower bound of 0.689, which represents the Pearson correlation coefficient between the S&P500 and the FTSE-MIB, and an upper bound of 0.9562 between CAC40 and DAX. Moreover, Fig. 1 emphasizes how most of the SARS-CoV-2 news refers to a specific interval around mid-March, in which most of the governments announced lockdown policies and consequent economic interventions. Notice also that the volatility series peak on March 11th, when the SARS-CoV-2 has been recognized as a pandemic by the World Health Organization (WHO 2020). Moreover, for sake of comparison, in the Appendix we report the analysis related to the impact and persistence volatility shocks induced by SARS-CoV-2 related news on the government bond market of the selected countries. The inclusion of country’s 1-Year bond yield allows us to determine which countries are the most efficient in incorporating SARS-CoV-2 shocks by characterizing their pre-shock and after-shock dynamics. From Fig. 6, notice that equity and bond markets react dissimilarly to major SARS-CoV-2 news. The equity indices volatility series show peaks on March 11th, whereas bond yields react to a greater extent to macroeconomic announcements such as the EU decision on the financial aid package worth 750 bln Euros to mitigate the damage of the coronavirus outbreak on the economy (March 23), and the IMF negative World Economic Outlook, released on April 14 (see IMF 2020).

**Fig. 2:**
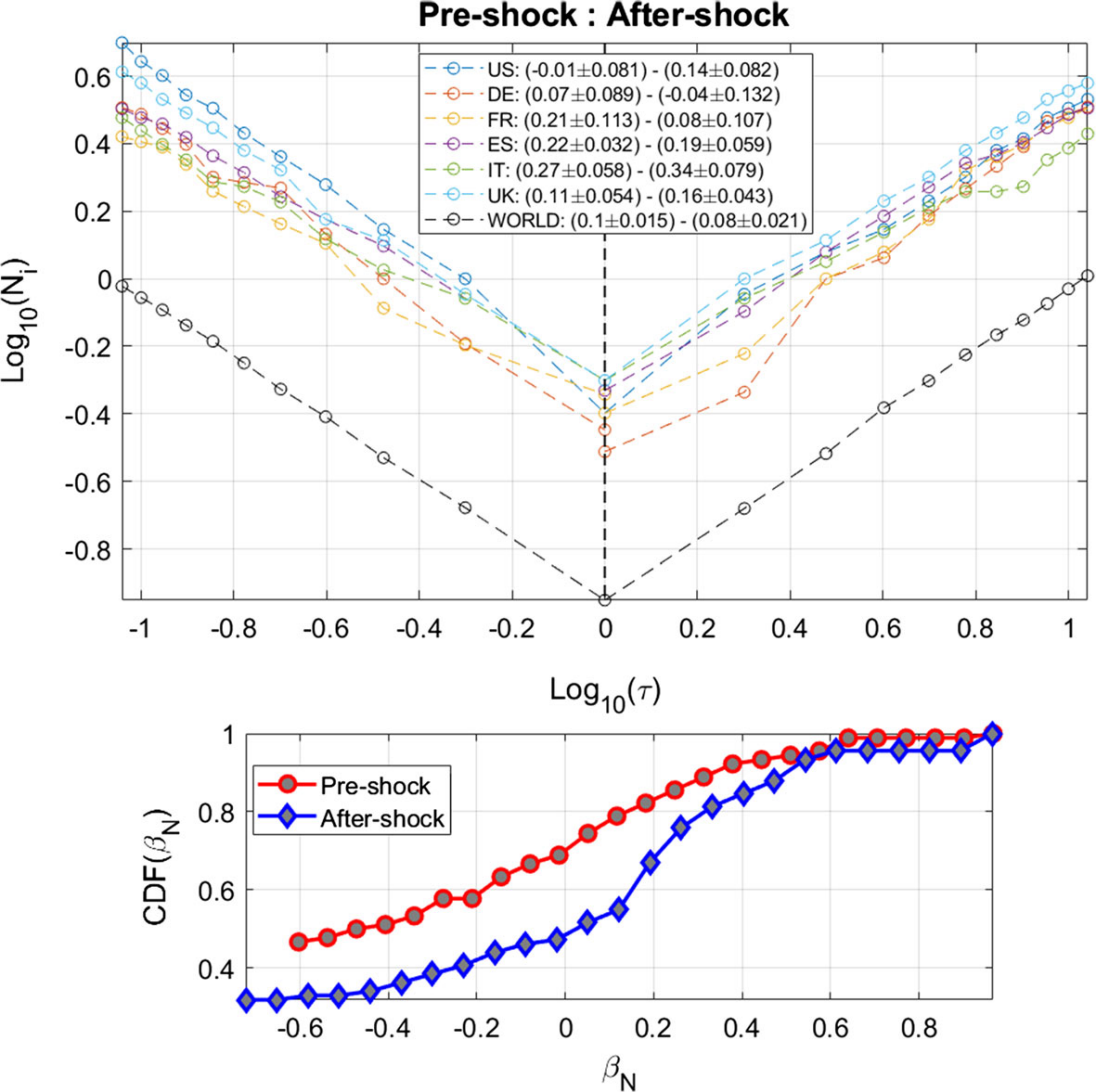
Average distribution of large volatility occurrences and Omori exponents. The upper panel reports the log-log plot of the average cumulative distribution of large volatility movements around the days of SARS-CoV-2 related announcements. Colored lines refer to the different financial markets while the black line corresponds to the SVD approximation of the aggregate volatility rates for the selected countries, labelled as WORLD in the figure’s legend. The legend provides the values of the Omori exponents for both pre-shocks and after-shocks. The lower panel shows the cumulative distributions of the pooled pre-shock and after-shock Omori exponents. In particular, the red color is used to identify the empirical CDF of the pre-shock exponents, while the blue color is associated with the after-shock distribution. (Color figure online)

To study the aggregate **equity** financial system reaction to the SARS-CoV-2 announcements, we approximate the market volatility through the first left-singular vector ${\mathbf {U}}$ obtained by the Singular Value Decomposition (SVD) of the volatility matrix ${\mathbf {V}}=[V_{1},\ldots ,V_{i}] \approx {\mathbf {U}}\Sigma {\mathbf {W}}$. We then proceed by studying the rate of occurrence of large fluctuations in such vector. The SVD proxy of the aggregate market volatility shows a peak located on March 11th and displays local maxima around the declaration of the Italian lockdown (March 8th) and the G7 finance ministers meeting of March 24th (see Appendix, Fig. 7).

Figure 2 describes the role played by SARS-CoV-2 announcements in generating large fluctuations in the considered financial indices, quantifying the volatility decay over time both before and after a market shock. In particular, the upper panel displays the average empirical cumulative distribution (CDF) of large volatility movements before and after the main SARS-CoV-2 announcements, together with the values of the Omori exponents for each market index, and for the SVD approximation of the aggregate volatility rate (indicated as WORLD in the figure’s legend). To determine the Omori power-law exponents and the corresponding confidence intervals, we employ a linear fit on a log-log scale.^4^ The Omori exponents can then be used to evaluate how markets react to SARS-CoV-2 related announcements. In particular, after the occurrence of a shock, large values of the Omori exponents indicate faster relaxation times, suggesting that the volatility generated by the announcements is rapidly reabsorbed by the market. On the contrary, low values of the Omori exponents stand for long-lasting effects on the volatility patterns, meaning that markets slowly return to their equilibrium state after perturbations induced by the SARS-CoV-2 announcements. Symmetrically, large Omori exponents can also be present in the pre-shock phase, meaning that a shock induces a sudden volatility jump near the date of the event, while low values of the exponents suggesting that the news are early discounted.

The considered financial markets present heterogeneous values of the Omori exponents. In particular, Italy, Spain and, to a lesser extent France, show the largest exponents, reflecting that the shocks effects are perceived shortly before the announcements and are absorbed in a timely manner. These countries refer to geographical areas which were first impacted by the SARS-CoV-2 contagion, and whose financial indices turned out to be more sensitive to SARS-CoV-2 related news. Conversely, Germany shows low Omori exponents, reflecting a more persistent impact of the SARS-CoV-2 news on its reference financial market, especially in the after-shock phase. Finally, the US and UK stock indices feature a mixed behavior: their volatility builds up slowly during the pre-shock, as for Germany, but then it is quickly reabsorbed in the after-shock phase, similarly to Spain. Moreover, the pre-shock Omori exponent computed on the left-singular vector of the SVD approximation of volatility matrix is larger than that of the corresponding after-shock, meaning that large volatility movements are absorbed more slowly.

Finally, the lower panel of Fig. 2 reports the empirical CDF of the Omori exponents computed by pooling together all the SARS-CoV-2 announcements for both the pre-shock and after-shock cases. The after-shock empirical CDF of $\beta _N$ exhibits larger values, with respect to the pre-shock distribution. Moreover, the two-sample Kolmogorov-Smirnov test rejects the null hypothesis that data refer to populations with the same distribution, with a p-value of about $10^{-5}$. Hence, larger values of $\beta ^{Aft}$, with respect to $\beta ^{Pre}$ confirm that the response time after $T_s$ is shorter than the activation time leading to $T_s$.

Moreover, as Fig. 9 in Appendix suggests, the bond yield series show an even more heterogeneous behaviour of the Omori exponents across countries. The magnitude of the pre-shock exponents is relatively high in Spain and, again to a lesser extent, in France if compared to the after-shock values. This suggests that the bond markets exhibit sudden volatility jumps just prior the news, which are then slowly reabsorbed. UK shows a similar behaviour only for the pre-shock phase. Interestingly, we also find a negative aftershock Omori exponent, although statistically significant only for the Italian and German bond volatility in the after-shock. This can be interpreted as a dominance of after-shocks further away from main-shocks over the volatility cascade around the event date. It is worth noting that, overall, bond yields exhibit lower Omori exponents than those observed for the equity indices: this suggests that the bond market is less efficient at incorporating SARS-CoV-2 related shocks. When considering bond yields, the difference between $\beta ^{Aft}$, and $\beta ^{Pre}$ is less prominent. This indicates that in the bond markets volatility induced by SARS-CoV-2 announcements is more persistent than that of equity markets, which instead react more timely to such exogenous shocks. This empirical outcome is in line with the studies on cross-market financial shock transmission, which find that volatility shocks in the equity markets are absorbed much more quickly than those in the bond markets (see, e.g., Tian and Hamori 2016).

We also report, in Fig. 10 of the Appendix, the Omori exponents related to each single SARS-CoV-2 announcement for both the pre-shock and after-shock cases, computed on the SVD representation of the aggregate volatility. We observe the presence of negative values for some $\beta ^{Pre}$, due to national lockdown declarations and ascribed to the market anticipation of these events. We also find negative values of $\beta ^{Aft}$ associated with announcements related to the first detected cases in Italy and to the approval of financial aid packages by the European Council: both these events turned out to contain a large amount of inherent surprise. Finally, Fig. 11 of the Appendix reports the sensitivity analysis of the Omori exponents computed by varying the parameter *q*. Notice that, by decreasing the threshold value, we increase the number of observations in the binary series associated with a high volatility, potentially leading to an overlapping of effects related to more events that contribute to volatility outbursts. This could translate in a switch in the sign of the Omori exponents since the CDF of the binary volatility would display a local maximum which is not attained in correspondence of the date of a precise event. This confounding effect can be ruled out by selecting a suitable higher threshold.

## Explaining the empirical evidence through agent-based modeling

The aim of this Section is to show how the dynamical interplay between agents’ behavior and stock price realizations is able to reproduce the observed empirical regularities of the considered financial indices. The activity of heterogeneous speculators is key to understand the observed market dynamics since the adjustment of their investment strategies in face of external shocks may generate long-lasting volatility patterns.

### Model building blocks

In recent years, Agent-Based models have brought a substantial progress in various areas of economic research (for surveys see, e.g., Tesfatsion and Judd 2006; Hens and Schenk-Hoppé 2009), especially in financial contexts for their capability of improving the understanding of the dynamics of financial markets (see Day and Huang 1990; Chiarella 1992; Lux 1995; Brock and Hommes 1998; LeBaron et al. 1999; Hommes and Wagener 2009; Bovi and Cerqueti 2016, among others). The dynamics of Agent-Based financial market models is driven by the trading behavior of heterogeneous speculators who follow trading strategies based on technical and fundamental analysis.

The model we propose (see, e.g., Lengnick and Wohltmann 2013; Westerhoff and Franke 2013) is not intended to quantitatively replicate the Omori exponents empirically observed, but rather to qualitatively show how market participants’ behavior could amplify and make persistent the volatility induced by the shocks. Since our main focus is on the financial effects of the SARS-CoV-2 announcements on the markets, we keep the Agent-Based framework as simple as possible to avoid confounding effects.

The model setup considers a market maker, who quotes an asset price with respect to the market excess demand, and heterogeneous speculators that determine their orders following a technical or a fundamental trading rule. According to the technical trading rule, prices move in trends and buying (selling) actions are suggested when prices increase (decrease). The fundamental trading rule predicts that the asset price will return towards its fundamental value. In undervalued markets, fundamental trading rules recommend buying while in overvalued markets they propose selling. Agents select one of the two trading strategies in each time period on the basis of an evolutionary mechanism which considers the past strategies performances (see Brock and Hommes 1997; Brock and Hommes 1998).

The details of the model are outlined as follows. The market maker collects all individual orders from trading agents and changes the price with respect to the resulting excess demand (see, e.g., Chiarella and He 2003; Hommes et al. 2005; Lengnick and Wohltmann 2013; Schmitt and Westerhoff 2017a; Ascari et al. 2018 for examples of models where the asset price is set according to the same price rule):

4 $$\begin{aligned} P_{t+1} = P_{t} + a(W_{t}^{c}D_{t}^{c}+W_{t}^{f}D_{t}^{f})+ \epsilon _{t} \end{aligned}$$

The variable *P* denotes the logarithm of the stock price, $D_{t}^{c}$ and $D_{t}^{f}$ represent the orders generated by chartists (*c*) and fundamentalists (*f*) respectively, $W_{t}^{c}$ and $W_{t}^{f}$ stand for the proportion of agents using these strategies, while *a* is a positive reaction parameter which we set equal to 1 without loss of generality. The noise term $\epsilon _{t}$ is i.i.d. normally distributed with zero mean and standard deviation $\sigma$, and represents an exogenous shock affecting price dynamics.

Chartists expect that the direction of the last observed price trend is going to persist, while fundamentalists expect that a fraction of the actual mispricing is corrected during the next period. Assuming that the demand generated by each type of investors depends positively on the expected price development, leads to:

5 $$\begin{aligned} \begin{aligned} D_{t}^{c} = l(P_{t}-P_{t-1})\\ D_{t}^{f} = g(F-P_{t}) \end{aligned} \end{aligned}$$

where *g*, *l* are positive reaction parameters while *F* is the fundamental value. The fractions of agents using the two different investment strategies are not fixed over time. Agents continuously evaluate the strategies according to their past performance. The better a strategy performs relative to the other, the more likely agents will employ it. Thus, the fraction of agents that adopts strategy $k=\{c,f\}$ is given by the discrete choice model (see, e.g., Manski and McFadden 1981):

6 $$\begin{aligned} W_{t}^{k}=\frac{\exp (eA_{t}^{k})}{\exp (eA_{t}^{c})+\exp (eA_{t}^{f})} \end{aligned}$$

where $A_{t}^{k}$ is the attractiveness of the *k*th strategy, which depends on its most recent performance as well as from its past attractiveness. This is formalized as:

7 $$\begin{aligned} A_{t}^{k}=(\exp (P_{t})-\exp (P_{t-1}))D^{k}_{t-2}+d A_{t-1}^{k}. \end{aligned}$$

The memory parameter $0 \le d \le 1$ defines the strength with which agents discount past strategy performances. The positive parameter *e* measures the intensity of choice. The higher (lower) *e*, the greater (lesser) the fraction of agents that will employ the strategy with the highest attractiveness. This switching mechanism is based on agents’ behavior: if the relative attractiveness of the fundamentalist strategy over the chartist one increases, the market share of chartists decreases and the market share of fundamentalists increases. In this sense, speculators exhibit a kind of learning behavior.

The baseline parameter values employed for the simulations are set according to Lengnick and Wohltmann (2013) as: $l=0.5$, $g=0.5$, $e=100$, $F=0$, $a=1$ and $d=0.5$. Moreover $\epsilon \sim {\mathcal {N}}(0,0.15)$.

### Agent-based simulations

**Fig. 3:**
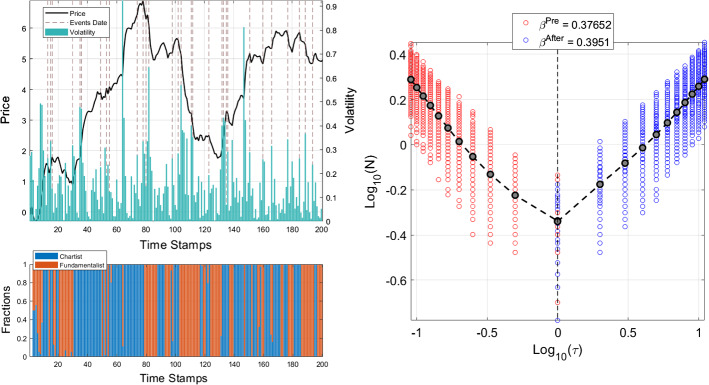
Price dynamics, volatility distributions and Omori exponents of the time series generated by the Agent-Based financial model. The upper left panel shows, in black, the price dynamics generated by a representative run of the heterogeneous agents model. Green bars refer to the related volatility, while dashed lines locate external shocks identified by extreme values of the noise term component in the price law of motion. The lower left panel reports the evolution of agents’ fraction adopting a chartist (blue) or a fundamentalist (orange) investment strategy. In the right panel, red and blue circles show, in log-log coordinates, the pre-shock and after-shock cumulative volatility distribution related to each model run, around the occurrence of the identified shocks. The black dashed line represents the average cumulative volatility distribution. The legend reports the values of the corresponding Omori exponents for both pre-shock and after-shock. (Color figure online)

As an illustrative example of stock price dynamics generated by the model, we report, in the upper left panel of Fig. 3, the price series resulting from a representative run, together with the corresponding volatility. The plot shows, in black, the price dynamics, while color bars refer to the related volatility. Moreover, the dashed lines identify the occurrence of extreme values of the noise term component and allows us to compute the volatility cumulative distribution both before and after such shock occurrences together with the related Omori exponents. Notice that the price law of motion in Eq. 4 is influenced at each time *t* by a random realization of the noise term $\epsilon _{t}$, which reflects the impact of external events. To select the time occurrence of the most relevant shocks in the model (which can play the same role of the SARS-CoV-2 announcements), we consider the realization of $\epsilon$ that lies above the 85-th percentile of the shock distribution. The lower left panel of Fig. 3 relates the price pattern to the evolution of the fractions of chartists (blue) and fundamentalists (orange) which populate the market. During periods in which technical traders dominate the market, high volatility patterns and bubbles may emerge. However, fundamental analysis becomes increasingly attractive as bubbles grow. If speculators switch from technical to fundamental analysis, then volatility decreases and prices gradually retreat towards their fundamental values.

Figure 3, right panel, shows the simulated CDF of large volatility occurrences along with the Omori exponents for both the pre-shock and after-shock. We run 1000 simulations of the model generating price time series of 200 points. For each realization, we identify the occurrence of extreme shocks and, around such points, we compute the empirical CDF of large volatility movements, as for the empirical series. Red and blue dots identify the cumulative distribution of each run, while the black dotted lines represent the average empirical CDFs, whose Omori exponents are reported in the box legend. The simulated volatility distribution displays a power-law behavior in line with the observed volatility, and the related Omori exponents have the same magnitude of those associated with the real financial indices.

### Sensitivity analysis

Our Agent-Based model allows us to investigate how the Omori exponents vary with respect to changes in agents’ investment attitudes, thus providing a behavioral rationale of the observed volatility dynamics. Since market participants can switch between trading strategies as well as respond to price changes with a different degree of reactivity, we investigate how such investment attitude produces dynamic relaxation in the volatility behavior in line with that occurring around SARS-CoV-2 related announcements. In particular, agents’ switching behavior is related to the intensity of the choice parameter *e* (see Brock and Hommes 1998), while chartists and fundamentalists reactivity to price changes are captured by the parameters *l* and *g*, respectively. We thus perform a sensitivity analysis by running the model on a 3D grid of 3000 points in the space $[50,150]\times [0.1,0.7]\times [0.1,0.7]$ corresponding to the parameters *e*, *l* and *g*, respectively, and recording, for each simulation, the values of the Omori exponents.

**Fig. 4:**
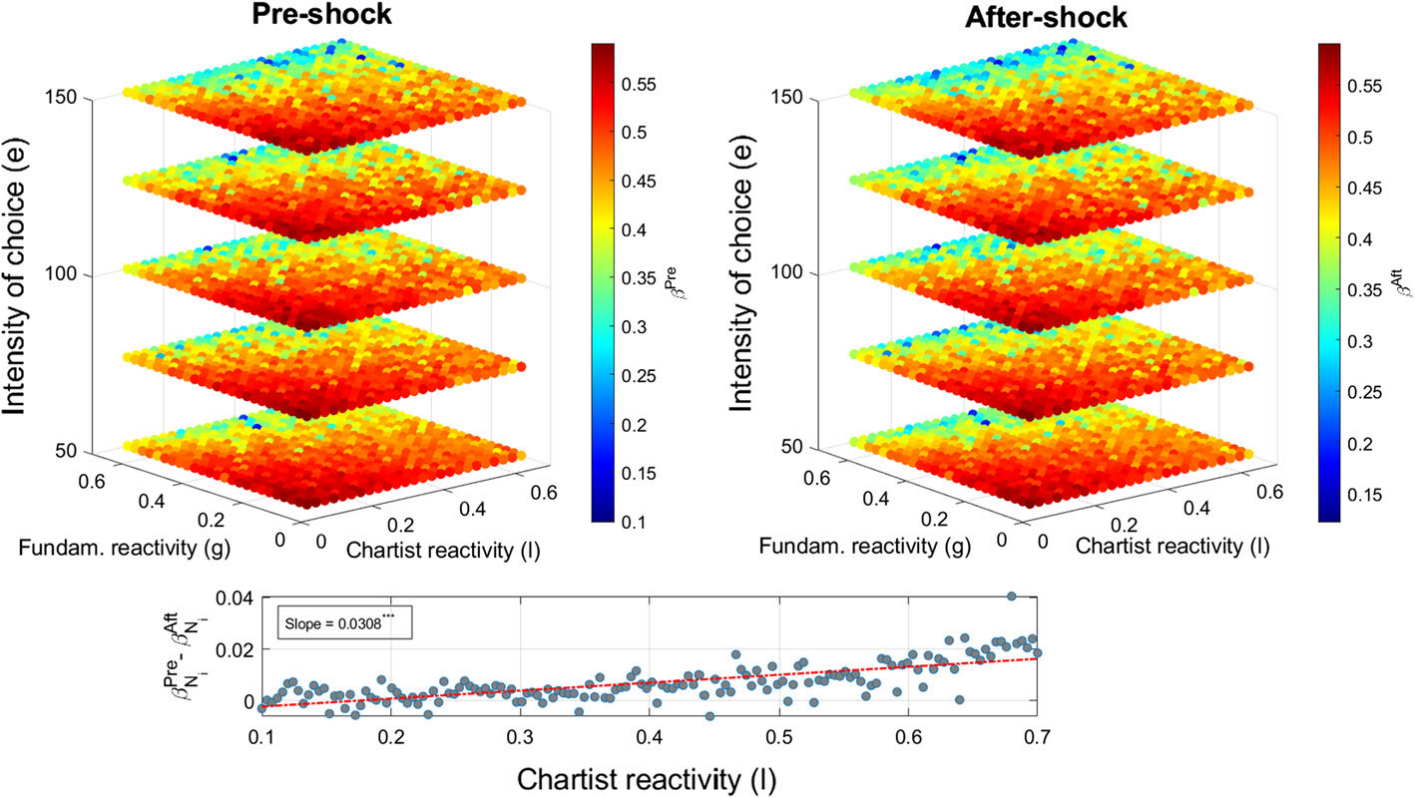
Omori exponents sensitivity to the model behavioral parameters. Upper panels report the pre-shock (left) and after-shock (right) Omori exponents on varying the market agents reactivity parameters, *l* and *g*, and the intensity of choice *e*. The color-bar maps colors into numerical values of Omori exponents. The lower panel displays the increasing relationship between the chartists reactivity parameter *l* and the difference between the pre-shock and after-shock exponents. The red dashed line represents the linear fitting whose slope parameter is reported in the annotation. (Color figure online)

Figure 4 reports the sensitivity analysis with respect to the model behavioral parameters. The left panel refers to the pre-shock case, while the right panel is associated with the after-shock case. The Omori exponents decrease (cold colors) as long as the intensity of choice *e* increases, since agent willingness to switch toward better performing strategies generates long-lasting price fluctuations. In the opposite case, when the intensity of choice parameter is relatively low, agents almost evenly distribute among strategies disregarding the performance measures. Thus, the occurrence of an exogenous shock induces a volatility outburst that is rapidly absorbed by the market since there is not a dominant trading strategy followed by the majority of the players. The absence of a continuous switching only leads to a temporary market perturbation. In addition, if agents’ reactivity parameters, *l* and *g*, are relatively weak, an exogenous shock hitting the market does not significantly alter agents’ demands and thus price realizations. In other words, again, the price is mostly influenced only at impact by the shock and displays a sudden volatility outburst which is rapidly reabsorbed. On the contrary, an increasing reactivity to price trend or to mispricing induces the emergence of marked and long-lasting bull and bear market phases through the asset demands, which create pronounced volatility patterns slowly decaying over time.

Finally, the lower panel of Fig. 4 presents intuitions on the economic mechanism which is at the ground of the asymmetry of the pre-shock and after-shock Omori exponents observed in the real data. This asymmetry can be associated to the different degree of agents’ reactivity. In particular, to qualitatively reproduce such stylized fact, we keep the parameter *g* fixed at 0.1, while we vary the chartists reactivity *l* form 0.1 to 0.7 generating 150 linearly distributed points in this space. For each parameter configuration, we run the model 1000 times. Each gray point represents the difference between the pre-shock and after-shock exponents generated by one run of the model, while the dashed red line identifies the linear fit with the corresponding slope reported in the inset. We observe a positive relationship between the chartists’ reactivity parameter *l* and the difference between the Omori exponents $\beta ^{Pre}_{N_{i}}-\beta ^{Aft}_{N_{i}}$. As long as the value of *l* increases, the after-shock induced volatility slower dissipates in the market. In fact, the volatility spike generated by the shock leads chartists to overreact to the price change at impact, making the asset price to deviate from its fundamental value. For this reason, some agents will start to believe that the price level is no longer sustainable and, therefore, they switch to the fundamental strategy. As a consequence, prices gradually retreat towards their fundamental values. Nonetheless, this condition reduces the attractiveness of the fundamental strategy leading directly to a new wave of chartism dominance. Whilst there is a permanent ongoing competition between the two trading rules, agents’ behavior is thus responsible for the long-lasting market volatility and long memory effects (see, e.g., Leal et al. 2016; Schmitt and Westerhoff 2017b).

## Concluding remarks

On Monday 24th February 2020, stock indices plunged in response to the SARS-CoV-2 outbreak, which has been spreading all over the world. The day after, financial markets remained wavy, reflecting hope that the economic fallout might be manageable. These markets’ movements mirror the uncertainty that prevails and persists on financial agents’ beliefs. Among economists, this ongoing situation more closely resembles the result of a natural disaster than a traditional economic recession (see Baker et al. 2020; Bram and Deitz 2020). Mattia Morandi, spokesman for Italy’s Ministry of Culture and Tourism, told The New York Times that this market phase “is seen as on par with an earthquake, a situation of emergency”. The economic contingency suddenly developed, in fact, as a consequence of a fast-moving global pandemic reflecting into large volatility movements in financial markets.

The SARS-CoV-2 pandemic has caused a worldwide financial distress and its impact on economic systems has proven to be heterogeneous and pervasive (see, e.g., Baker et al. 2020; Bonaccorsi et al. 2020; Spelta et al. 2020b; IMF 2020; McKee and Stuckler 2020; OECD 2020). In this work, we have proposed to assess the market impact of SARS-CoV-2 announcements through the Omori law by quantifying the rate of occurrence of large volatility movements deriving parallels between energy dissipation, as observed for earthquakes, and financial fluctuations. We have analysed data from 6 different financial systems in a multivariate perspective, differently from what has been usually done in literature, with the exception of Selçuk (2004) that focused on emerging markets. The joint consideration of several markets has allowed us to compare different responses and, therefore, better understand market reactions to extreme events. This, in turn, can reveal insights for the economic outlook, for the evaluation of effectiveness of containment efforts and for the related recovery patterns.

Not unlike an earthquake, the market response to the SARS-CoV-2 news generates volatility rates that decay over time as a power-law, here interpreted as market inefficiency in processing the flow of information. In particular, financial markets have shown a different degree of reaction to the events that hit their respective country, being them either surprised or able to early discount the effect of the related news. Besides quantifying the after-shock market response, we have also uncovered the presence of quantifiable pre-shocks effects. We have observed that countries earlier impacted by the SARS-CoV-2 contagion feature the largest exponents, reflecting that the effects of shocks are perceived shortly before the announcements and are rapidly absorbed. On the contrary, more capitalized financial indices, such as DAX, FTSE100 and the S&P500, respond weakly to the shocks generated by the SARS-CoV-2 announcements. Hence, if one can observe part of the dataset, then by fitting the data by the Omori law with estimated parameters, it is possible to predict how long the relaxation will take and how large effects it will bring under the hypothesis of power-law relaxation. Thus, such information would be useful to plan a recovery and investments into the financial markets. Our results are also of potential interest for traders which operate on a short time scale. Indeed, the statistical regularity found for the different financial markets, before and after a market shock, could be used for hedging purposes, since the Omori response dynamics provides a time window over which after-shocks can be expected. In general, rare extreme events such as market crashes constitute a substantial risk for investors. Due to the scaling properties of the Omori law, it is possible to analyze the statistics of the consequent volatility cascades for different thresholds by studying the behavior of the consequent occurring fluctuations.

In the second part of our work, through a stylized Agent-Based model we have shown how agents’ behavior could amplify the market dynamics induced by the shocks, generating long-lasting volatility patterns. The willingness of speculators to switch toward better performing investment strategies, as well as their reactivity to the price trend, are key to understand the observed market underreaction (see Barberis et al. 1998). This finding implies that markets take a finite time to re-adjust prices following announcements and relate the market response, quantified by the Omori exponents, to the change in the agents’ expectations before and after the shocks.

Overall, our analysis not only bears relevance for the application of the seismologic approach to extreme events that hit different financial markets, but it also contains a further element that has not been considered before in the literature, namely the understanding of how the markets’ response depend on the behaviour of financial traders. On one hand, the choice of the seismologic approach is coherent with the fact that, after a large market crash, markets show long-lasting activities in line with the Omori law. On the other hand, the long-lasting price dynamics may be due to the interacting behavior of market’s participants that can herd towards a common strategy and that lead the price to deviate from its fundamental value. This feature may cause crashes, and thus volatility outbursts, to be locally self-enforcing or dissipating.

In conclusion, we can state that in a context characterized by complex behaviors due to the financial crises generated by the SARS-CoV-2 pandemic, addressing short-term shocks in the financial system will not be enough to face acute market risks derived from SARS-CoV-2. A further level of analysis can thus be required. The adoption of a stylized Agent-Based framework turns out to be relevant for the understanding of investors behavior at a micro-level reacting to different policy scenarios implemented at a macro-level, and to undertake the emerging challenges that are rapidly engulfing businesses affected by the pandemic.

## Figures and Tables

**Fig. 5:**
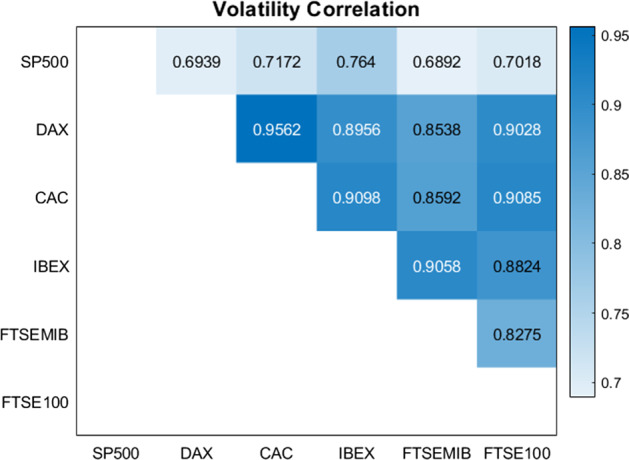
Financial indices volatility correlation. The figure shows the pairwise Pearson correlation coefficients among the volatility series of the considered financial indices. Darker colors correspond to higher correlated pairs. (Color figure online)

**Fig. 6:**
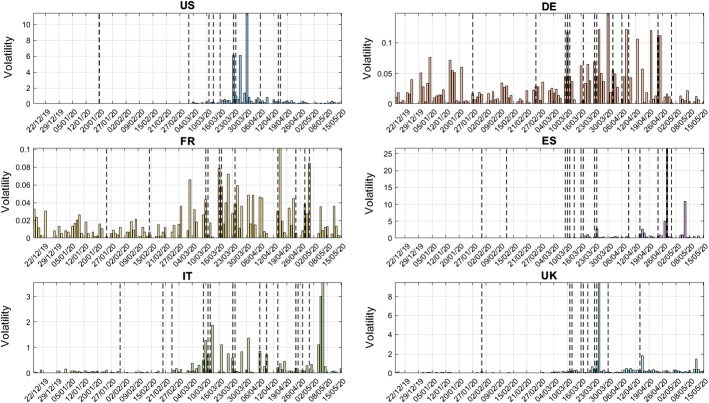
Volatility patterns for selected bond yields along with the events dates. Column bars represent, for each country, the daily volatility of the reference bond yields (lower panel). The dashed black lines identify the dates of the relevant events, which mainly impacted the course of the national bond markets

**Fig. 7:**
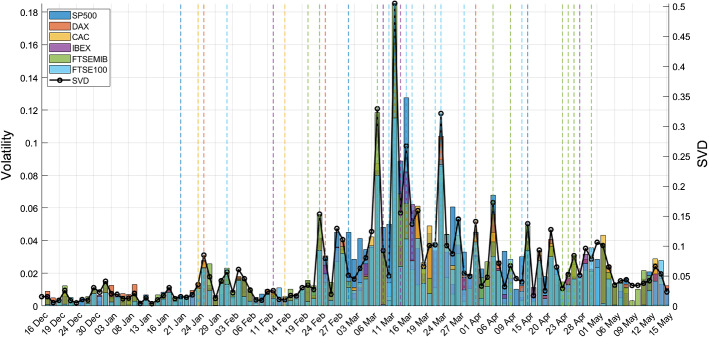
SVD proxy of the aggregate volatility for the selected countries. The black solid line proxies the aggregate volatility dynamics obtained through the SVD of the volatility matrix ${\mathbf {V}}=[V_{1},\ldots ,V_{i}] \approx {\mathbf {U}}\Sigma {\mathbf {W}}$. Colored bars refer to the daily volatility of the different market indices while dashed colored lines identify the days of SARS-CoV-2 related events in each country. Colors are associated to the different markets according to the legend. (Color figure online)

**Fig. 8:**
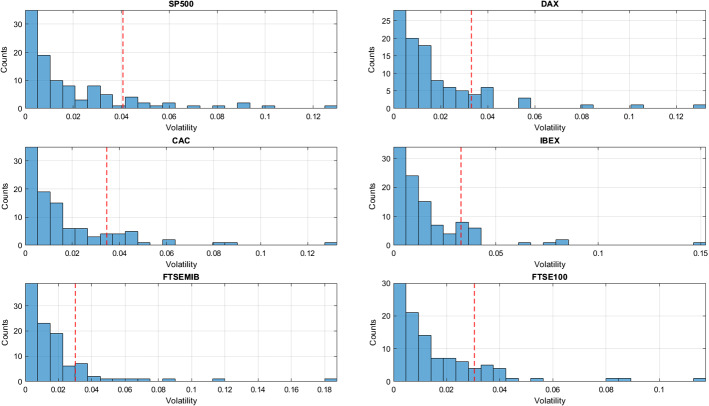
Volatility distributions. The figure reports the volatility distributions for the reference financial markets highlighting with red dashed bars the 85th percentiles taken as a reference for identifying the threshold for high volatility movements. (Color figure online)

**Fig. 9:**
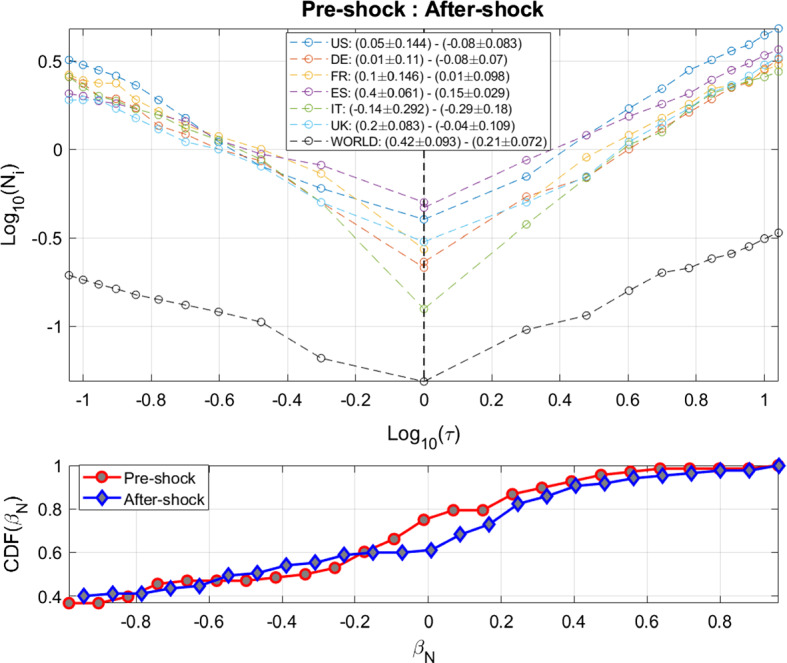
Average distribution of large volatility occurrences and Omori exponents for the bond markets. The upper panel report the log-log plot of the average cumulative distribution of large volatility movements around the days of SARS-CoV-2 related announcements. The legend provides the values of the Omori exponents for both pre-shocks and after-shocks. The lower panels show the cumulative distributions of the pooled pre-shock and after-shock Omori exponents. In particular, the red color is used to identify the empirical CDF of the pre-shock exponents, while the blue color is associated with the after-shock distribution. (Color figure online)

**Fig. 10:**
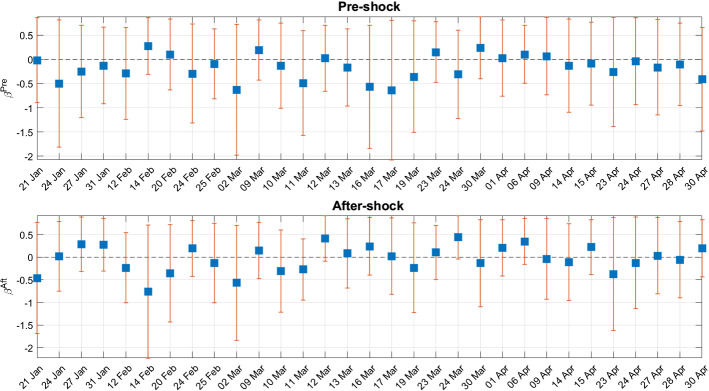
Omori exponents for each single SARS-CoV-2 event computed on SVD proxied volatility. The figure shows the Omori exponents related to the cumulative aggregate volatility distribution around the single dates of SARS-CoV-2 related news. The upper panel refers to the pre-shock case while the lower panel displays the exponents for the after-shock case

**Fig. 11:**
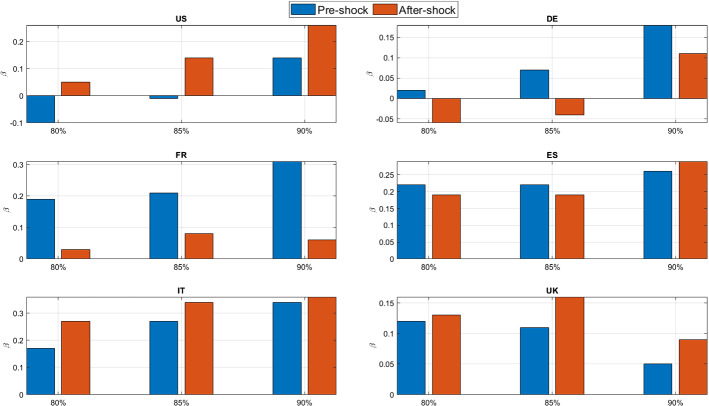
Omori exponents for different thresholds of the volatility distribution. The figure shows the Omori exponents related to the cumulative aggregate volatility distribution and computed by varying the parameter *q*, which is the percentile of the volatility distribution used for defining the binary volatility series

## Appendix

See Tables 1 and 2 and Figs. 5, 6, 7, 8, 9, 10 and 11.

**Table 1:** SARS-CoV-2 related events for USA, Germany and France

*United States*	
20/01/2020	First confirmed case
29/02/2020	First reported death
11/03/2020	The World Health Organization’s Director-General declares that SARS-CoV-2 can be characterized as a pandemic
13/03/2020	Approval of an aid economic package for workers and individuals
16/03/2020	Trump issues guidelines to avoid social gatherings and to restrict discretionary travels
22/03/2020	Trump announces the approval of Washington emergency declaration
24/03/2020	The White House and Senate leaders of both parties announced agreement of a $\S$2 trillion measure to aid workers,
	businesses and the healthcare system
06/04/2020	The Federal Reserve announces it will support banks that lend to small businesses
14/04/2020	The International Monetary Fund estimates global GPD to decline of about 3%
15/04/2020	Trump announces guidelines on reopening the US economy
*Germany*	
25/02/2020	First confirmed cases in the Baden-Württemberg region
09/03/2020	First reported death
10/03/2020	Merkel announces up to 70% of Germany could become infected
11/03/2020	Merkel announces liquidity support for companies
	The World Health Organization’s Director-General declares that SARS-CoV-2 can be characterized as a pandemic
17/03/2020	The health threat switches from moderate to high
23/03/2020	The government decides on a financial aid package of 750 billion
24/03/2020	The European Commission approves, under the Temporary Framework, a German scheme to support companies
	G7 finance ministers and central bank governors meeting, pledging to do “whatever is necessary” to help their
	economies recover from the coronavirus
01/04/2020	Social distancing measures are extended until April 19th
09/04/2020	The ministers of Finances of the Eurozone countries agreed to a 500 billions aid, including the possibility of using
	the European Stability Mechanism
14/04/2020	The International Monetary Fund estimates global GPD to decline of about 3%
23/04/2020	The European Council approves a financial aid package worth 540 billions
30/04/2020	The European Central Bank announces new pandemic emergency longer-term refinancing operations
*France*	
24/01/2020	First confirmed case
14/02/2020	First reported death
10/03/2020	Introduction of mobility and activities restrictions
11/03/2020	The World Health Organization’s Director-General declares that SARS-CoV-2 can be characterized as a pandemic
16/03/2020	Announcement of national lockdown
17/03/2020	The French Finance Minister Le Maire announces a 45 billions aid package for small businesses and other hard-hit sectors
24/03/2020	The European Commission approves, under Article 107(3)(b), three French State aid schemes
	G7 finance ministers and central bank governors meeting, pledging to do “whatever is necessary” to help their
	Economies recover from the coronavirus
14/04/2020	The International Monetary Fund estimates global GPD to decline of about 3%
23/04/2020	The European Council approves a financial aid package worth 540 billions
28/04/2020	The Prime Minister reveals plans to ease SARS-CoV-2 lockdown measures
30/04/2020	The European Central Bank announces new pandemic emergency longer-term refinancing operations

**Table 2:** SARS-CoV-2 related events for Spain, Italy and United Kingdom

*Spain*	
31/01/2020	First confirmed case
12/02/2020	First reported death
09/03/2020	State of emergency declaration in the community of Madrid
10/03/2020	The European Commission proposes to “free up 7.5 billions of liquidity”
11/03/2020	The World Health Organization’s Director-General declares that SARS-CoV-2 can be characterized as a pandemic
13/03/2020	The state of emergency is extended to the whole country
15/03/2020	Declaration of national lockdown
17/03/2020	Announcement of a 200 billions support package
22/03/2020	Lockdown measures are extended until April 11th
24/03/2020	G7 finance ministers and central bank governors meeting, pledging to do “whatever is necessary” to help their
	Economies recover from the coronavirus
09/04/2020	The ministers of Finances of the Eurozone countries agree to a 500 billions aid, including the possibility of using
	the European Stability Mechanism
13/04/2020	Adoption of some gradual measures for easing the lockdown
14/04/2020	The International Monetary Fund estimates global GPD to decline of about 3%
23/04/2020	The European Council approves a financial aid package worth 540 billions
28/04/2020	Unveiling of a gradual exit strategy from lockdown
30/04/2020	The European Central Bank announces new pandemic emergency longer-term refinancing operations
*Italy*	
31/01/2020	First confirmed cases in Rome (Chinese couple)
20/02/2020	First confirmed case in Codogno
22/02/2020	First death in Veneto and creation of the first “red zones” in Lombardy and Veneto
09/03/2020	Declaration of national lockdown
10/03/2020	The European Commission proposes to “free up 7.5 billions of liquidity”
11/03/2020	Further restrictions on lockdown related to travel and approval of a 25 billions financial package
	The World Health Organization’s Director-General declares that SARS-CoV-2 can be characterized as a pandemic
21/03/2020	Halt to all non-essential production activities
24/03/2020	G7 finance ministers and central bank governors meeting, pledging to do “whatever is necessary” to help their
	economies recover from the coronavirus
06/04/2020	Announcement of an economic stymulus worth 200 billions
09/04/2020	The ministers of Finances of the Eurozone countries agreed to 500 billion aid, including the possibility of using the ESM
14/04/2020	The International Monetary Fund estimates global GPD to decline of about 3%
23/04/2020	The European Council approves a financial aid package worth 540 billions
24/04/2020	Conversion of the “Cura Italia” decree into law
26/04/2020	New prime minister decree on the beginning of the “phase 2” for the reopening of economic activities and the easing
	Of mobility restrictions
30/04/2020	The European Central Bank announces new pandemic emergency longer-term refinancing operations
*United Kingdom*	
31/01/2020	First confirmed cases
11/03/2020	The World Health Organization’s Director-General declares that SARS-CoV-2 can be characterized as a pandemic
12/03/2020	The Chief Medical Officers raises the risk for the UK from moderate to high
16/03/2020	Prime Minister Johnson advises everyone against non-essential travel and contact with others
17/03/2020	Chancellor Sunak announces that $\pounds$330 billions would be made available in loan guarantees for businesses
	affected by the pandemic
19/03/2020	The government introduces the Coronavirus Bill 2019–2021
23/03/2020	Prime Minister Johnson announces that measures to mitigate the virus will to be tightened further
24/03/2020	G7 finance ministers and central bank governors meeting, pledging to do “whatever is necessary” to help their
	Economies recover from the coronavirus
28/03/2020	Fitch downgrades the UK to AA-
14/04/2020	The Office for Budget Responsibility publishes a scenario under which UK GDP would fall by 35%
	in the second quarter of 2020. The International Monetary Fund estimates global GPD to decline of about 3%

## Footnotes

1 https://www.who.int/docs/default-source/coronaviruse/situation-reports/20200420-sitrep-91-covid-19.pdf?sfvrsn=fcf0670b_4.

2 Even if China has been the first country hit by the pandemic, it has not been selected due to the scarcity of news and difficulty in acquiring political and economical announcements.

3 We would like to point out that Lillo and Mantegna (2003, 2004) demonstrate how, after a market crash, the volatility is represented by a stochastic process characterized by a power-law decay rate. Therefore, autoregressive models, such as GARCH models, are less able to describe the observed behavior since these models are characterized by an exponential decay.

4 We have employed 10-base logarithm scale for analogy with seismology. Indeed, the number of after-shocks after an earthquake has been demonstrate to be linearly dependent on displacement time when plotted in 10-base logarithm scale (Omori 1894).
